# Complete genome sequence of *Weeksella virosa* type strain (9751^T^)

**DOI:** 10.4056/sigs.1603927

**Published:** 2011-02-22

**Authors:** Elke Lang, Hazuki Teshima, Susan Lucas, Alla Lapidus, Nancy Hammon, Shweta Deshpande, Matt Nolan, Jan-Fang Cheng, Sam Pitluck, Konstantinos Liolios, Ioanna Pagani, Natalia Mikhailova, Natalia Ivanova, Konstantinos Mavromatis, Amrita Pati, Roxane Tapia, Cliff Han, Lynne Goodwin, Amy Chen, Krishna Palaniappan, Miriam Land, Loren Hauser, Yun-Juan Chang, Cynthia D. Jeffries, Evelyne-Marie Brambilla, Markus Kopitz, Manfred Rohde, Markus Göker, Brian J. Tindall, John C. Detter, Tanja Woyke, James Bristow, Jonathan A. Eisen, Victor Markowitz, Philip Hugenholtz, Hans-Peter Klenk, Nikos C. Kyrpides

**Affiliations:** 1 DSMZ - German Collection of Microorganisms and Cell Cultures GmbH, Braunschweig, Germany; 2 DOE Joint Genome Institute, Walnut Creek, California, USA; 3 Los Alamos National Laboratory, Bioscience Division, Los Alamos, New Mexico USA; 4 Biological Data Management and Technology Center, Lawrence Berkeley National Laboratory, Berkeley, California, USA; 5 Lawrence Livermore National Laboratory, Livermore, California, USA; 6 HZI – Helmholtz Centre for Infection Research, Braunschweig, Germany; 7 University of California Davis Genome Center, Davis, California, USA; 8 Australian Centre for Ecogenomics, School of Chemistry and Molecular Biosciences, The University of Queensland, Brisbane, Australia

**Keywords:** strictly aerobic, slimy, Gram-negative, lyses proteins, inhabitant of mucosa, *Flavobacteriaceae*, GEBA

## Abstract

*Weeksella virosa* Holmes *et al.* 1987 is the sole member and type species of the genus *Weeksella* which belongs to the family *Flavobacteriaceae* of the phylum *Bacteroidetes*. Twenty-nine isolates, collected from clinical specimens provided the basis for the taxon description. While the species seems to be a saprophyte of the mucous membranes of healthy man and warm-blooded animals a causal relationship with disease has been reported in a few instances. Except for the ability to produce indole and to hydrolyze Tween and proteins such as casein and gelatin, this aerobic, non-motile, non-pigmented bacterial species is metabolically inert in most traditional biochemical tests. The 2,272,954 bp long genome with its 2,105 protein-coding and 76 RNA genes consists of one circular chromosome and is a part of the *** G****enomic* *** E****ncyclopedia of* *** B****acteria and* *** A****rchaea * project.

## Introduction

Strain 9751^T^ (= DSM 16922 = NCTC 11634 = JCM 21250) is the type strain of *Weeksella virosa,* which is the sole member and type species of the genus *Weeksella* [1,2]. The generic name was given in honor of O.B. Weeks for his contributions to the taxonomy of the genus *Flavobacterium*. The species epithet is derived from the Latin word ‘virosa’ meaning ‘slimy’, referring to the colony appearance of the species [1]. *W. virosa* strain 9751^T^ was isolated from a clinical specimen of urine and described by Holmes and coworkers in 1986 [1]. These authors collected 29 strains from clinical samples, mostly obtained in the USA but also in other nations and continents, as the basis for their species description. Most isolates came from genitourinary tract samples, predominantly from women. Since then, strains of *W. virosa* were detected by cultural methods in the oral cavity [3], the genitourinary tract [4,5] of  man, in clinical specimens of pigs [6], the urine of a cow with bladder carcinoma [7], and in the midgut of a Brazilian dipteran [8]. *Weeksella*-like strains were also found in food samples [9] and environmental samples, however, the latter matched the genus description but could not be assigned to the species *virosa* thus suggesting that they are representatives of additional species within the genus that are not yet described [10,11]. About 2% of healthy women carry *W. virosa* on their vaginal mucosa [4]. Two cases of peritonitis have been described as being caused by *W. virosa* [12,13]. Here we present a summary classification and a set of features for *W. virosa* 9751^T^, together with the description of the complete genomic sequencing and annotation.

## Classification and features

A representative genomic 16S rRNA sequence of strain 9751^T^ was compared using NCBI BLAST under default settings (e.g., considering only the high-scoring segment pairs (HSPs) from the best 250 hits) with the most recent release of the Greengenes database [14] and the relative frequencies, weighted by BLAST scores, of taxa and keywords (reduced to their stem [15]) were determined. The five most frequent genera were *Chryseobacterium* (45.9%), *Wautersiella* (23.2%), *Myroides* (7.9%), *Elizabethkingia* (7.3%) and *Empedobacter* (7.0%) (134 hits in total). Regarding the single hit to sequences from members of the species, the average identity within HSPs was 99.2%, whereas the average coverage by HSPs was 96.8%. Among all other species, the one yielding the highest score was *Empedobacter brevis*, which corresponded to an identity of 92.0% and an HSP coverage of 97.0%. Accordingly, *E. brevis* groups as the sister genus in trees constructed from 16S rRNA gene sequences [16-19] whereas *Bergeyella zoohelcum* (family *Flavobacteriaceae*) is looked at as the phenotypic sister taxon which may be confused with *W. virosa* when applying traditional morphological and physiological tests [11]. The highest-scoring environmental sequence was GQ383925 ('sewage water isolate XJ109 *Flavobacteriaceae* str. XJ109'), which showed an identity of 92.5% and a HSP coverage of 98.5%. The five most frequent keywords within the labels of environmental samples which yielded hits were 'skin' (11.2%), 'human' (3.7%), 'fossa' (3.4%), 'microbiom, tempor, topograph' (2.8%) and 'forearm' (2.1%) (116 hits in total). The two most frequent keywords within the labels of environmental samples which yielded hits of a higher score than the highest scoring species were 'sewag, water' (10.1%) and 'aerosol, air, citi, level, microarra, texa, two, urban' (10.0%) (2 hits in total). From these sequence-based observations and the published culture-dependent detections (see above) it can be concluded that *W. virosa* occurs mainly as an inhabitant of mucosa of animals and man.

**Figure 1:**
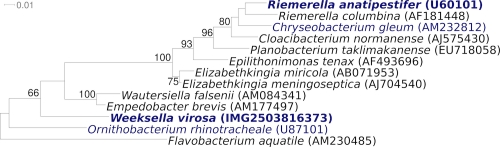
Phylogenetic tree highlighting the position of *W. virosa* relative to a selection of closely related other type strains within the family *Flavobacteriaceae*. The tree was inferred from 1,339 aligned characters [20,21] of the 16S rRNA gene sequence under the maximum likelihood criterion [22] and rooted in accordance with the type strain of the family. The branches are scaled in terms of the expected number of substitutions per site. Numbers above branches are support values from 700 bootstrap replicates [13] if larger than 60%. Lineages with type strain genome sequencing projects registered in GOLD [23] are shown in blue, published genomes in bold. The genome of *Riemerella anatipestifer* is described in this issue of SIGS [24].

Figure 1 shows the phylogenetic neighborhood of strain 9751^T^ in a 16S rRNA based tree. The sequences of the five 16S rRNA gene copies in the genome differ from each other by up to three nucleotides, and differ by up to two nucleotides from the previously published 16S rRNA sequence (M93152), which contains eleven ambiguous base calls, and differ by up to 314 nucleotides from another previously published 16S rRNA sequence (AF133539) which obviously represents a strain of the genus *Bacteroides*.

**Figure 2:**
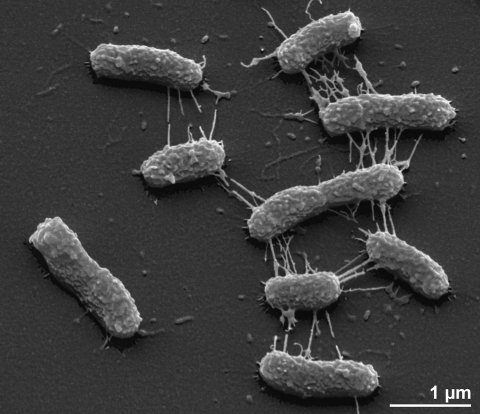
Scanning electron micrograph of *W. virosa* strain 9751^T^

The cells of *W. virosa* are rod-shaped (0.6 × 2.0-3.0 µm) with parallel sides and rounded ends (Figure 2). According to Holmes [1], the cells stain Gram-negative, are nonmotile and non spore-forming (Table 1). No intracellular inclusions such as polyhydroxybutyrate were detected. Colonies on nutrient agar appear circular and low convex with entire edges, smooth, shining and mucoid, reaching 2mm in diameter after 24h [1]. While the colonies are described as being non-pigmented on nutrient agar by most authors [1,37], the production of a nondiffusible yellow pigment was reported during growth on blood agar [5]. Most strains produce a diffusible dark brown pigment on tyrosine-containing agar [1]. The strains grow at 42°C but not at 5°C [1]. *W. virosa* is a strictly aerobic chemoorganotroph and is not able to reduce nitrate, nitrite or selenite and does not acidify glucose or other sugars under standard conditions [1,5]. However, under test conditions developed for fastidious organisms such as *Neisseria* (API NH strips, Biomérieux), acid is produced from glucose [IDA] as is observed  for the phylogenetic neighbors *E. brevis* and *Wautersiella falsenii* [16,17]. Cytochrome oxidase, catalase and phosphatase are present [1]. Tolerance to NaCl and pH ranges have not been reported. *W. virosa* grows on McConkey agar, indicating tolerance to bile salts [1] . On the other hand, KCN (75 mg l^-1^), cetrimide, colistin [17], and polymyxin [5] are not tolerated and inhibit growth. The species is able to utilize β-hydroxybutyrate as a substrate and to hydrolyze casein, gelatin and Tween 20 [1,17]. Tryptophan is cleaved to give indole, pyruvate and ammonia when tested with Ehrlich’s reagent, but the reaction can not be detected when Kovacs’ reagent is used [1] . *W. virosa* is inert in most traditional biochemical tests [1], it does not utilize glucose as a substrate under standard conditions  [16]. *W. virosa* does not hydrolyze starch, esculin or DNA, and is negative for gluconate oxidation, urease, phenylalanine deaminase, arginine deaminase, arginine dihydrolase, lysine or ornithine decarboxylase, β-D-galactosidase [1], alkalization of galacturonate [17]. The following enzymes are present as concluded from tests using API ZYM galleries: acid and alkaline phosphatase, lysine arylamidase, aspartate arylamidase, alanine arylamidase and methionine arylamidase [1]. In addition, the following substrates were hydrolyzed in the latter galleries by *W. virosa*: naphthol-AS-BI-phosphodiamide, bis-(para-nitrophenyl)-phosphate, glycyl-glycyl-β-naphthylamide hydrobromide, glycyl-L-phenylalanyl-β-naphthylamide, glycyl-L-prolyl-β-naphthylamide, L-leucyl-glycyl-β-naphthylamide, α-L-glutamyl-β-naphthylamide, and N-carbobenzoxy-glycyl-glycyl-L-arginine-β-naphthylamide [1]. *W. virosa* is susceptible to most β-lactams, tetracycline, chloramphenicol, nalidixic acid, erythromycin and sulfamethoxazole-trimethoprim [11]. In contrast, the species is resistant to aminoglycosides [11].

**Table 1:** Classification and general features of *W. virosa* 9751^T^ according to the MIGS recommendations [25].

MIGS ID	Property	Term	Evidence code
	Current classification	Domain *Bacteria*	TAS [26]
		Phylum *Bacteroidetes*	TAS [27]
		Class ‘*Flavobacteria*’	TAS [28]
		Order ‘*Flavobacteriales*’	TAS [29]
		Family *Flavobacteriaceae*	TAS [30-33]
		Genus *Weeksella*	TAS [1,34]
		Species *Weeksella virosa*	TAS [1,34]
		Type strain 9751 (= CL345/78)	TAS [1,34]
	Gram stain	negative	TAS [1]
	Cell shape	rod-shaped	TAS [1]
	Motility	non-motile	TAS [1]
	Sporulation	none	TAS [1]
	Temperature range	10°C or less up to 42°C	TAS [1]
	Optimum temperature	not reported	NAS
	Salinity	not reported	NAS
MIGS-22	Oxygen requirement	aerobic	TAS [1]
	Carbon source	proteins	TAS [1]
	Energy source	chemoorganotroph	TAS [1]
MIGS-6	Habitat	human urogenital tract	TAS [1,4]
MIGS-15	Biotic relationship	saprophyte	TAS [1,11]
MIGS-14	Pathogenicity	none	NAS
	Biosafety level	1	TAS [35]
	Isolation	human urine	TAS [1]
MIGS-4	Geographic location	North Carolina, USA	TAS [1]
MIGS-5	Sample collection time	1986 or before	TAS [1]
MIGS-4.1	Latitude	not reported	NAS
MIGS-4.2	Longitude	not reported	NAS
MIGS-4.3	Depth	not reported	NAS
MIGS-4.4	Altitude	not reported	NAS

Evidence codes - IDA: Inferred from Direct Assay (first time in publication); TAS: Traceable Author Statement (i.e., a direct report exists in the literature); NAS: Non-traceable Author Statement (i.e., not directly observed for the living, isolated sample, but based on a generally accepted property for the species, or anecdotal evidence). These evidence codes are from of the Gene Ontology project [36]. If the evidence code is IDA, then the property was directly observed by one of the authors or an expert mentioned in the acknowledgements.

### Chemotaxonomy

The major respiratory quinone of *W. virosa* is menaquinone 6 and the major polyamine is homospermidine, as is the case for all members of the family *Flavobacteriaceae* [11,38-40]. No sphingophospholipids were detected [1]. The polar lipids of *W. virosa* have not yet been described. The major whole-cell fatty acids of *W. virosa* are *iso*-C_15:0_ (46%), *iso*-C_15:0_2-OH (10%), *iso*-C_17:1_ω12t (8%) and *iso*-C_17:0_3-OH (7%) as described for CDC group IIf, the preliminary name given to these strains prior to being formally named *W. virosa* [41]. A comparison of the patterns of *W. virosa* and ‘*W. zoohelcum*’ obtained at that time [41] with more recently published patterns of *B. zoohelcum* and *E. brevis* and phylogenetic neighbors [17,19] seems to cast doubts on the comparability of these early patterns. They are the only ones listing the presence of high amounts of *iso*-C_15:0_2-OH and *iso*-C_17:1_ω12t, which are not listed for phylogenetically related genera later on [19]. However, *iso*-C_15:0_2-OH and isomers of *iso*-heptadecene are included in the summed features of the Microbial Identification System applied in many recent analyses including [17,19].

## Genome sequencing and annotation

### Genome project history

This organism was selected for sequencing on the basis of its phylogenetic position [42], and is part of the *** G****enomic* *** E****ncyclopedia of* *** B****acteria and* ***  A****rchaea * project [40]. The genome project is deposited in the Genomes OnLine Database [23] and the complete genome sequence is deposited in GenBank. Sequencing, finishing and annotation were performed by the DOE Joint Genome Institute (JGI). A summary of the project information is shown in Table 2.

**Table 2:** Genome sequencing project information

**MIGS ID**	**Property**	**Term**
MIGS-31	Finishing quality	Finished
MIGS-28	Libraries used	Tree genomic libraries: one 454 pyrosequence standard library, one 454 PE library (17 kb insert size), one Illumina library
MIGS-29	Sequencing platforms	Illumina GAii, 454 GS FLX Titanium
MIGS-31.2	Sequencing coverage	2,107.5 × Illumina; 64.3 × pyrosequence
MIGS-30	Assemblers	Newbler version 2.5-internal-10Apr08-1-threads, Velvet version 0.7.63, phrap
MIGS-32	Gene calling method	Prodigal 1.4, GenePRIMP
	INSDC ID	CP02455
	Genbank Date of Release	February 15, 2011
	GOLD ID	Gc01619
	NCBI project ID	50581
	Database: IMG-GEBA	2503754024
MIGS-13	Source material identifier	DSM 16922
	Project relevance	Tree of Life, GEBA

### Growth conditions and DNA isolation

*W. virosa* 9751^T^, DSM 16922, was grown on DSMZ medium 220 (Caso Agar) [37] at 30°C. DNA was isolated from 0.5-1 g of cell paste using MasterPure Gram-positive DNA purification kit (Epicentre MGP04100) following the standard protocol as recommended by the manufacturer, with modification st/DL for cell lysis as described in Wu *et al*. [43]. DNA is available through the DNA Bank Network [44,45].

### Genome sequencing and assembly

The genome was sequenced using a combination of Illumina and 454 sequencing platforms. All general aspects of library construction and sequencing can be found at the JGI website [46]. Pyrosequencing reads were assembled using the Newbler assembler version 2.5-internal-10Apr08-1-threads (Roche). The initial Newbler assembly consisting of 27 contigs in one scaffold was converted into a phrap assembly by making fake reads from the consensus, to collect the read pairs in the 454 paired end library. Illumina GAii sequencing data (4,788 Mb) was assembled with Velvet [47] and the consensus sequences were shredded into 1.5 kb overlapped fake reads and assembled together with the 454 data. The 454 draft assembly was based on 131.6 Mb 454 draft data and all of the 454 paired end data. Newbler parameters are -consed -a 50 -l 350 -g -m -ml 20. The Phred/Phrap/Consed software package [48] was used for sequence assembly and quality assessment in the subsequent finishing process. After the shotgun stage, reads were assembled with parallel phrap (High Performance Software, LLC). Possible mis-assemblies were corrected with gapResolution, Dupfinisher [49], or sequencing cloned bridging PCR fragments with subcloning or transposon bombing (Epicentre Biotechnologies, Madison, WI). Gaps between contigs were closed by editing in Consed, by PCR and by Bubble PCR primer walks (J.-F.Chang, unpublished). A total of 60 additional reactions were necessary to close gaps and to raise the quality of the finished sequence. Illumina reads were also used to correct potential base errors and increase consensus quality using a software Polisher developed at JGI [50]. The error rate of the completed genome sequence is less than one in 100,000. Together, the combination of the Illumina and 454 sequencing platforms provided 2,171.8 × coverage of the genome. The final assembly contained 384,925 pyrosequence and 63,008,730 Illumina reads.

### Genome annotation

Genes were identified using Prodigal [51] as part of the Oak Ridge National Laboratory genome annotation pipeline, followed by a round of manual curation using the JGI GenePRIMP pipeline [52]. The predicted CDSs were translated and used to search the National Center for Biotechnology Information (NCBI) nonredundant database, UniProt, TIGR-Fam, Pfam, PRIAM, KEGG, COG, and InterPro databases. Additional gene prediction analysis and functional annotation was performed within the Integrated Microbial Genomes - Expert Review (IMG-ER) platform [53].

## Genome properties

**Figure 3:**
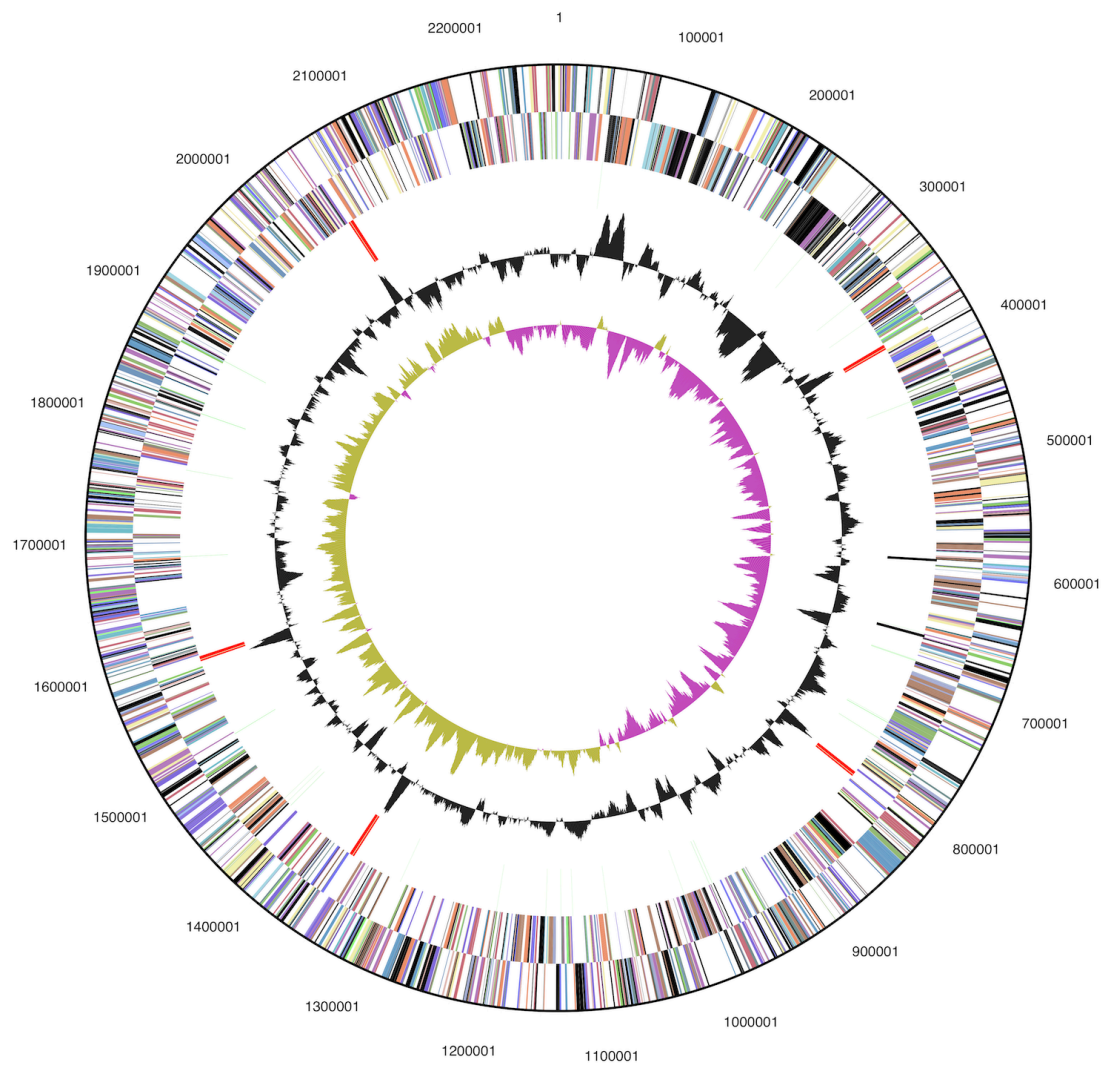
Graphical circular map of the chromosome. From outside to the center: Genes on forward strand (color by COG categories), Genes on reverse strand (color by COG categories), RNA genes (tRNAs green, rRNAs red, other RNAs black), GC content, GC skew.

The genome consists of a 2,272,954 bp long chromosome with a GC content of 35.9% (Table 3 and Figure 3). Of the 2,181 genes predicted, 2,105 were protein-coding genes, and 76 RNAs; 56 pseudogenes were also identified. The majority of the protein-coding genes (65.5%) were assigned with a putative function while the remaining ones were annotated as hypothetical proteins. The distribution of genes into COGs functional categories is presented in Table 4.

**Table 3:** Genome Statistics

**Attribute**	**Value**	**% of Total**
Genome size (bp)	2,272,954	100.00%
DNA coding region (bp)	2,037,648	89.65%
DNA G+C content (bp)	816,484	35.92%
Number of replicons	1	
Extrachromosomal elements	0	
Total genes	2,181	100.00%
RNA genes	76	3.48%
rRNA operons	5	
Protein-coding genes	2,105	96.52%
Pseudo genes	56	2.57%
Genes with function prediction	1,429	65.52%
Genes in paralog clusters	99	4.54%
Genes assigned to COGs	1,403	64.33%
Genes assigned Pfam domains	1,534	70.33%
Genes with signal peptides	506	23.20%
Genes with transmembrane helices	451	20.68%
CRISPR repeats	1	

**Table 4:** Number of genes associated with the general COG functional categories

Code	value	%age	Description
J	142	9.5	Translation, ribosomal structure and biogenesis
A	0	0.0	RNA processing and modification
K	54	3.6	Transcription
L	94	6.3	Replication, recombination and repair
B	0	0.0	Chromatin structure and dynamics
D	18	1.2	Cell cycle control, cell division, chromosome partitioning
Y	0	0.0	Nuclear structure
V	29	1.9	Defense mechanisms
T	29	1.9	Signal transduction mechanisms
M	154	10.3	Cell wall/membrane/envelope biogenesis
N	4	0.3	Cell motility
Z	0	0.0	Cytoskeleton
W	0	0.0	Extracellular structures
U	25	1.7	Intracellular trafficking, secretion, and vesicular transport
O	73	4.9	Posttranslational modification, protein turnover, chaperones
C	82	5.5	Energy production and conversion
G	47	3.1	Carbohydrate transport and metabolism
E	127	8.5	Amino acid transport and metabolism
F	57	3.8	Nucleotide transport and metabolism
H	95	6.3	Coenzyme transport and metabolism
I	72	4.8	Lipid transport and metabolism
P	101	6.7	Inorganic ion transport and metabolism
Q	24	1.6	Secondary metabolites biosynthesis, transport and catabolism
R	171	11.4	General function prediction only
S	103	6.9	Function unknown
-	778	35.7	Not in COGs
